# Side-population cells in luminal-type breast cancer have tumour-initiating cell properties, and are regulated by HER2 expression and signalling

**DOI:** 10.1038/sj.bjc.6605553

**Published:** 2010-02-09

**Authors:** T Nakanishi, S Chumsri, N Khakpour, A H Brodie, B Leyland-Jones, A W Hamburger, D D Ross, A M Burger

**Affiliations:** 1 Departments of Medicine, Pathology, Pharmacology and Experimental Therapeutics, University of Maryland, School of Medicine, Marlene and Stewart Greenebaum Cancer Center (UMGCC), Baltimore, MD, USA; 2 Department of Hematology and Medical Oncology, Winship Cancer Center, Emory University, Atlanta, GA, USA; 3 Baltimore VA Medical Center, Baltimore, MD, USA; 4 Barbara Ann Karmanos Cancer Institute and Department of Pharmacology, Wayne State University, Detroit, MI, USA

**Keywords:** SP, BC, luminal, HER2, T-ICs

## Abstract

**Background:**

The expression of side-population (SP) cells and their relation to tumour-initiating cells (T-ICs) have been insufficiently studied in breast cancer (BC). We therefore evaluated primary cell cultures derived from patients and a panel of human BC cell lines with luminal- or basal-molecular signatures for the presence of SP and BC stem cell markers.

**Methods:**

The SPs from luminal-type BC were analysed for BC T-IC characteristics, including human epidermal growth factor receptor 2 (HER2), ER*α*, IGFBP7 expression and their ability to initiate tumours in non-obese diabetic severe combined immunodeficiency (NOD/SCID) mice. Pharmacological modulators were used to assess the effects of HER2 signalling and breast cancer-resistance protein (BCRP) expression on SPs.

**Results:**

The SP was more prevalent in the luminal subtype of BC compared with the basal subtype. HER2 expression was significantly correlated with the occurrence of an SP (*r*^2^=0.75, *P*=0.0003). Disappearance of SP in the presence of Ko143, a specific inhibitor of the ATP-binding cassette transporter BCRP, suggests that BCRP is the predominant transporter expressed in this population. The SP also decreased in the presence of HER2 signalling inhibitors AG825 or trastuzumab, strengthening the notion that HER2 contributed to the SP phenotype, likely through downstream AKT signalling. The SP cells from luminal-type MCF-7 cells with enforced expression of HER2, and primary cells with luminal-like properties from a BC patient, displayed enrichment in cells capable of repopulating tumours in NOD/SCID mice. Engraftment of SP cells was inhibited by pretreatment with AG825 or by *in vivo* treatment with trastuzumab.

**Interpretation:**

Our findings indicate an important role of HER2 in regulating SP and hence T-ICs in BC, which may account for the poor responsiveness of HER2-positive BCs to chemotherapy, as well as their aggressiveness.

According to the stem cell hypothesis, tumour-initiating cells (T-ICs) are the only population capable of recapitulating the original tumour. In breast cancer (BC), a CD44^+^/CD24^–low^/Lin^−^ cell population was first identified as T-ICs (Al-Hajj *et al*, 2003). Later, aldehyde dehydrogenase (ALDH) 1 activity was reported to be associated with stem/progenitor properties in BC (Ginestier *et al*, 2007). More recent studies showed that these T-IC populations in BC might be regulated through human epidermal growth factor receptor (HER) 2. In patients with HER2-positive tumours receiving the epidermal growth factor receptor (EGFR)/HER2 inhibitor lapatinib in an adjuvant setting, a decrease in CD44^+^/CD24^−/low^ cells and tumour mammosphere-forming efficiency was observed. The ALDH1^+^ T-ICs were increased by upregulation of ‘stemness’ genes through HER2 overexpression in BC (Korkaya *et al*, 2008; Li *et al*, 2008; Magnifico *et al*, 2009). However, ALDH1^+^ T-ICs were more frequently detected in the ‘triple-negative’ (HER2-, oestrogen-, and progesterone receptor-negative) basal-like-subtype BC than in luminal BC (Charafe-Jauffret *et al*, 2009), suggesting that T-ICs may differ with the subtype of BC.

Compared with CD44^+^/CD24^−low^/Lin^−^ and ALDH1^+^ T-ICs, the effect of HER2 on side-population (SP) T-ICs has not been well studied (Hirschmann-Jax *et al*, 2004; Ponti *et al*, 2005). The SPs from BC contain primitive stem cell-like cells that can differentiate into epithelial tumours *in vitro* and *in vivo* and express stemness genes (Patrawala *et al*, 2005; Christgen *et al*, 2007). As SP cells have a high drug efflux capacity owing to functional expression of ABC transporters such as BCRP (ABCG2) and P-glycoprotein (Pgp/ABCB1) (Hirschmann-Jax *et al*, 2004), T-ICs in the SP may be more relevant to drug resistance than CD44^+^/CD24^−low^/Lin^−^ and ALDH1^+^ T-ICs. In fact, HER2 is a predictive marker for responses to drugs that are effluxed through BCRP and/or Pgp, including cyclophosphamide, methotrexate and fluorouracil, (Menard *et al*, 2001; Wolff *et al*, 2007), docetaxel (Xie *et al*, 2008), as well as endocrine therapies, particularly tamoxifen (Konecny *et al*, 2003). Therefore, the activation of drug-resistant SP cells could provide an explanation for the poor response of HER2-positive and luminal-type tumours to cytotoxic chemotherapy.

A previous study of head and neck squamous cell carcinoma indicated an increase in SP cells by activation of HER1/EGFR signalling (Chen *et al*, 2006). Furthermore, the stimulatory effect of EGF on BCRP gene transcription has been reported in human breast (Meyer zu Schwabedissen *et al*, 2006) and ovarian cancer (Nakanishi *et al*, 2006b). Hence, we investigated whether HER2 could be a key regulatory factor for T-ICs found in the SP of BC. Furthermore, we evaluated whether HER2 can affect the engraftment of SP cells from primary and BC cell lines in non-obese diabetic severe combined immunodeficiency (NOD/SCID) mouse repopulation assays. In this study, we show that the SP is prevalent and enriched for T-ICs in the luminal-subtype BC, and that HER2 can expand T-ICs in primary and permanent BC cells. Our data support a pivotal role for HER2 in regulating SP cells in luminal-type BC.

## Materials and methods

### Cell culture

Human BC cell lines (MCF10A, MCF-7, T47D, MDA-MB-231, Hs578T, MDA-MB-468, SKBR3, and BT-20) were obtained from the American Type Culture Collection (Manassas, VA, USA). The MCF-7 cell line transfected with human *HER2*, MCF-7/HER2-18, was generated and maintained as previously described (Jerome *et al*, 2006). MDA-MB-231 and BT-20 cells were transfected with either the pcDNA3 vector (Invitrogen, Carlsbad, CA, USA) containing human HER2 cDNA or with pcDNA3 alone. Transfectants were selected using geneticin, and individual colonies were screened for HER2 expression by western blots. Established *HER2*-overexpressing MDA-MB-231 and BT-20 cells were designated as MDA-MB-231/HER2 and BT-20/HER2, respectively. All cell lines were cultured in RPMI 1640 medium (Mediatech Inc., Manassas, VA, USA) containing 10% foetal bovine serum. Aromatase-transfected MCF-7 cell lines (Ac1) and anastrozol-resistant Ac1 (Ac1ANAR) were cultured as previously described (Zhou *et al*, 1990). Primary cell cultures were established as outlined in Supplemental Methods; they were designated as GCC-BC1 to 4, an abbreviation for Greenebaum Cancer Center-Breast Cancer line number 1–4; their characteristics are listed in Supplementary Table S1.

### SP and fluorescence-activated cell sorting analyses

The SP protocol was essentially performed as described by Goodell *et al* (1996). Cells (1 × 10^6^ cells per ml) were incubated in Dulbecco's Modified Eagle's Medium containing 5% foetal bovine serum, 10 mM HEPES, and 5 *μ*g ml^–1^ Hoechst 33342 (H33342, from Sigma-Aldrich, St Louis, MO, USA) for 90 min at 37 °C with or without BCRP inhibitor Ko143 (1 *μ*M, a gift from Dr Schellens, Netherlands Cancer Institute). Samples were analysed by flow cytometry (LSR I or FACSVantage SE, BD Biosciences, San Jose, CA, USA, Supplementary Figure S1). For isolation of SP cells from BC cell lines or from primary cultures, the FACSVantage SE cell sorter was used. At least 10^7^ cells were subjected to fluorescence-activated cell sorting to yield sufficient SP cells for *in vitro* and *in vivo* experiments.

Allophycocyanin-, fluorescein isothiocyanate-, or phycoerythrin-labelled primary antibodies for phenotyping of SP cells for surface antigens included antibodies to BCRP (R&D Systems Inc., Minneapolis, MN, USA), HER2 (R&D Systems), Pgp, CD44, or CD24 (from Chemicon, Gibbstown, NJ, USA). Propidium iodide (2 *μ*g ml^–1^; Sigma-Aldrich) was added to discriminate dead cells. For the determination of CD44^+^/CD24^−^ and ALDH1^+^ cells, 2–5 × 10^5^ cells were suspended in 50 *μ*l of 0.5% bovine serum albumin in phosphate-buffered saline (PBS), and were then incubated with allophycocyanin-conjugated anti-CD44, fluorescein isothiocyanate-conjugated anti-CD24, or with both istotype controls as described before (Al-Hajj *et al*, 2003; Hughes *et al*, 2008). The ALDH1^+^ cells were analysed using ALDEFLUOR (StemCell Technologies, Vancouver, BC, Canada) according to the manufacturer's instructions. Data were processed using WinMDI software version 2.8 (http://facs.scripps.edu/software.html).

### Clonogenic assay

The human tumour clonogenic assay was performed as described by us earlier (Fiebig *et al*, 2004). In brief, to test *in vitro* tumour cell colony-forming ability as a surrogate of self-renewal, 5000–10 000 cells were seeded per well in a 24-well plate and grown until colonies reached a diameter between 50 and 200 *μ*m. Plating efficiency was determined as % of cells forming a colony per number of cells seeded.

### Western blotting

Cells were washed with ice-cold PBS and homogenised in RIPA buffer by sonication as previously described (Nakanishi *et al*, 2006c). A volume of 50 *μ*g of cell lysate was subjected to 10% SDS–polyacrylamide gel electrophoresis. Blots were then probed with anti-BCRP (Sigma-Aldrich), anti-HER2/c-Neu (EMD Chemicals, Gibbstown, NJ, USA), anti-phosphorylated HER2 (Tyr 1221/1222), anti-HER3, anti-phosphorylated HER3 (Tyr 1289), anti-AKT, anti-phosphorylated AKT (Ser 473), anti-phospho p38 mitogen-activated protein kinase (MAPK) (Thr180/Tyr182)(12F8) (rabbit mAb #4631, Cell Signaling Technology Inc., Beverly, MA, USA), and anti-*β* actin (Sigma). The blots were developed with horseradish peroxidase-conjugated secondary antibodies (Santa Cruz Biotechnologies, Santa Cruz, CA, USA) and Millipore chemiluminescence reagent (Fisher Scientific, Pittsburgh, PA, USA). Signals were developed with Kodak Biomax films, Buffalo, NY, USA and signal intensities were analysed relative to *β* actin, using NIH ImageJ software (Bethesda, MD, USA).

### NOD/SCID mouse repopulation assay

All animal experiments were performed according to a protocol approved by the University of Maryland Institutional Animal Care and Use Committee (IACUC). The IACUC at the University of Maryland follows the guidelines of UKCCCCR for the welfare of animals and experimental neoplasia (Workman *et al*, 1998). A volume of 100 *μ*l of culture medium mixed with 100 *μ*l of Matrigel (BD Biosciences) containing 100,500 or 1000 sorted SP cells was either transplanted immediately into the mammary gland of 6-week-old female NOD/SCID/Ncr mice (Animal Production Facility, NCI Frederick, MD, USA) by subcutaneous injection, or incubated with 100 *μ*M AG825 (Sigma-Aldrich) for 2 h before injection. Trastuzumab was obtained from our hospital pharmacy and administered intraperitoneally twice weekly at a dose of 8 mg kg^–1^ as previously described (Jerome *et al*, 2006). As a control for enrichment of T-ICs in SP cells, whole cell population cells were injected at dilutions of 1000, 10 000, and 5 × 10^6^ into NOD/SCID mice. Tumour growth was followed up weekly by palpation and monitored for 6 months or until a size of ⩽1500 mm^3^ was reached (the limit defined by our IACUC end-point criteria). The number of tumours >100 mm^3^ was counted over the same observation period. On termination of experiments, animals were killed and tumours were excised and subjected to immunohistochemistry.

### Immunocytology

Sorted SP cells were fixed on glass slides by immersing thrice for 1 min each in ice-cold methanol/acetone (v/v 1 : 1). The slides were blocked with 5% bovine serum albumin in PBS overnight at 4 °C and incubated for 2 h with anti-ER*α* (clone 6F11, Novacastra, Newcastle upon Tyne, UK, 1 : 200 in PBS), anti-IGFBP7 antibody (1 : 100 in PBS, Burger *et al*, 1998), cytokeratin 18 (CK18), or anti-HER2 antibodies (Cell Signaling), according to the manufacturer's instructions. Immunofluorescence was visualised with goat anti-mouse fluorescein isothiocyanate- or anti-rabbit TRITC-conjugated secondary antibodies (Sigma; 1 : 400, 3 h), and then counterstained with 4′-6-diamidino-2-phenylindole (2 mg ml^–1^; Sigma-Aldrich). Immunoperoxidase staining for IGFBP7 and ER*α* was performed as reported before (Burger *et al*, 1998). Results were documented using a Leica DM4000 microscope with Improvision Openlab software (Wetzlar, Germany).

### Statistical analysis

Wilcoxon or Student's *t*-tests were used to assess the significance of the difference between the two means of data resulting from *in vitro* assays. The Spearman's rank coefficient test was used for correlation analyses. The analysis of variance F-test was used to analyse the significance of the *in vivo* tumour repopulation data. The software packages used were SPSS SYSTAT version 10 (SYSTAT Software, Chicago, IL, USA) and the statistics module of Microsoft Office Excel (version 2003).

## Results

### T-IC type and transcriptional profiles of BC cells

**Figure 1:**
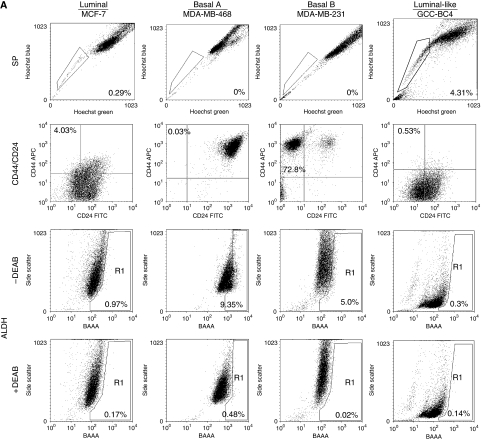

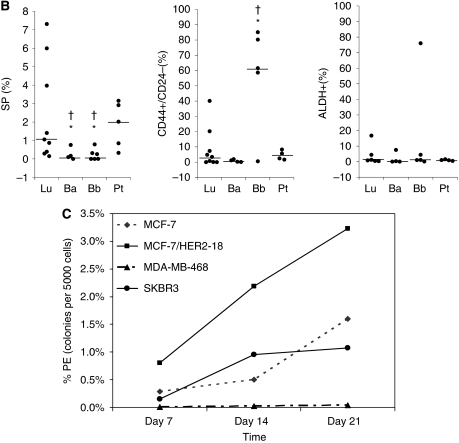
Association of stem cell markers with transcriptional classification of breast cancer (BC) cells. (**A**) The side-population (SP) cells were analysed in MCF-7, MDA-MB-231, MDA-MB-468, and GCC-BC4 cells by Hoechst staining and flow cytometry. To determine CD44^+^/CD24^−^ expression, cells were incubated with anti-CD44 (conjugated with allophycocyanin (APC)) and anti-CD24 (conjugated with fluorescein isothiocyanate (FITC)), or both isotype controls. Aldehyde dehydrogenase 1^+^ (ALDH1^+^) was analysed by measuring cellular fluorescence of bodipy-aminoacetate (BAAA) in the presence or absence of DEAB, a specific inhibitor for ALDH1. Percentages of cell fractions positive for stem cell markers are shown in the quadrants of the graphs containing the relevant cell population. Each plot is representative of at least three independent experiments. (**B**) Analysis of breast tumour-initiating cells (T-ICs) defined as SP, CD44^+^/CD24^−^, or ALDH1^+^ cell fractions. Twenty-five different human BC cell lines were evaluated. Average values of each surrogate markers for T-ICs in a given cell line were plotted in dot plots. Lu BC includes 11 cell lines: MCF-7, MCF-7/human epidermal growth factor receptor 2 (HER2)-18, MCF-7/vector, HC-7, SKBR3, T47D, MCF-7/TAM1, MCF-7 CA, MCF-7 CA/LTLT (Sabnis *et al*, 2009), Ac1, and Ac1/ANAR; Ba BC includes four lines: BT-20, BT-20/pcDNA3, BT-20/HER2, and MDA-MB468; Bb BC includes six lines: MCF10A, MDA-MB231, MDA-MB231/pcDNA3, MDA-MB231/HER2, Hs578T, and Hs578Ti8 (Hughes *et al*, 2008). Because of a lack of transcriptional profiling data for the patient-derived BC lines, these cells were classified as luminal-like and include GCC-BC1, -BC2, -BC3, and -BC4. The median percentage values are indicated by a horizontal bar. Lu, Luminal: Ba, Basal A; Bb, Basal B; Pt, Patient-derived primary cell line. ^*^P<0.01 *vs* Lu BC; ^†^*P*<0.01 *vs* Pt BC, by Wilcoxon test. (**C**) Clonogenic assay for representative cells from panel B. The % plating efficiency (PE) representing colony-forming units in the whole cell population per 5000 seeded cells was highest in cell lines with large SP, such as MCF-7/HER2-18, and lowest in those BC cells lacking an SP (MDA-MB-468). The data shown represent the mean of three independent experiments.

Surrogate markers for breast T-ICs (CD44^+^/CD24^−^, ALDH1^+^ and SP) were determined by fluorescence-activated cell sorting in a panel of cultured BC cells with luminal (Lu) or basal (B) global transcriptome expression profiles (Neve *et al*, 2006). The Lu subtype mostly represents oestrogen receptor/ER-positive BCs that may or may not be progesterone receptor/PR- and/or HER2-positive tumours, whereas the B subtype lacks the expression of these receptors, and is often designated ‘triple-negative’ (Andre and Pusztai, 2006). Neve *et al* further subdivided basal-like BC cell lines into Ba and Bb. This classification has so far not been carried out with BC tissue; instead, the latter has been subclassified into luminal-A, luminal-B, and luminal-C categories (Andre and Pusztai, 2006). According to Neve *et al* (2006), the Ba subtype is positive for cytokeratin 5 and 14; Bb is vimentin positive. Both Ba and Bb exhibit a stem-like expression profile that reflects the clinical triple-negative tumour type. The classifications of the BC cell lines used in this study are shown in Table 1. Comparative T-IC marker analyses are shown in Figure 1A for the MCF-7, MDA-MB-468, and MDA-MB-231 lines, representing Lu, Ba, and Bb subtypes, respectively. GCC-BC1–4 cells, for which gene expression analysis has not yet been performed, were considered to be Lu-like because of ER and CK18 expression (Supplementary Table S1, Supplementary Figure S3). Interestingly, the prevalence of T-IC markers among different subtypes of BC cells was different (Figure 1A, Table 1), with SP being present in Lu-type or Lu-like cells and low or absent in Ba/Bb-type cells.

Dot plots show the median value for the presence of breast T-IC markers for each BC subtype (Figure 1B). Patient-derived cell lines (Pt) were listed separately because of lack of a definitive gene cluster analysis (Figure 1B, Table 1). The median value of the percentage of SP cells in a given whole cell population was the highest in Lu-subtype cells (1.09%), which was 11-fold higher than that in Ba-subtype cells (*P*<0.01). The SP cells were lowest in Bb-type cells. Whereas SP cells were mainly present in Lu-subtype cancers (e.g., MCF-7 and GCC-BC4), ALDH1^+^ cells were detected in Lu and Ba/Bb-subtype cells such as MDA-MB-468 and MDA-MB-231 (Figure 1A, B). The median values of ALDH1^+^ cells were almost identical in Lu-subtype (0.81%) and patient-derived cells (0.82%). Although the Ba-subtype MDA-MB-468 cells had the highest ALDH1^+^ fraction (7.56%, Table 2, Figure 1B), the mean percentage value of ALDH1^+^ cells in the Ba subtype (0.35%) was the lowest among all gene clusters, because ALDH1 was not detected in other Ba BCs, such as BT-20 cells (0.02%). A large population of CD44^+^/CD24^−^ cells (80.3 and 56.7%, respectively) was characteristic for highly metastatic Bb-subtype BC cells (median value=72.1%) including MDA-MB-231 or Hs578T (Hughes *et al*, 2008). Importantly, all three different subtypes of BC T-ICs were increased in MCF-7/HER2-18 cells compared with those in parental MCF-7 cells (Table 2). In addition to a greater than 10-fold increase in SP cells in MCF-7/HER2-18 described above, CD44^+^/CD24^−^ and ALDH1^+^ cells were also increased 1.53- and 3.55-fold, respectively (Table 2). In addition, we performed clonogenic assays as *in vitro* tests for self-renewal (Fiebig *et al*, 2004) in a panel of cell lines from Tables 1 and 2; the percentage plating efficiency representing colony-forming units in the whole cell population was highest in cell lines with a large SP, such as MCF-7/HER2-18, and lowest in those BC cells lacking an SP and HER2, expressing low levels of CD44^+^/CD24^−^, but exhibiting a high ALDH1^+^ fraction (MDA-MB-468) (Figure 1C). Overall, these data are in support of a regulatory role of HER2 in expansion of cells with T-IC properties.

### HER2 expression and the SP in BC cells

**Figure 2:**
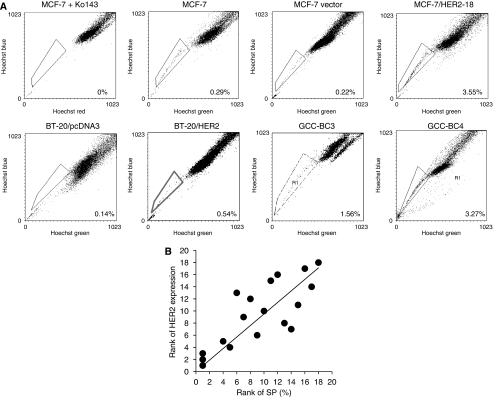

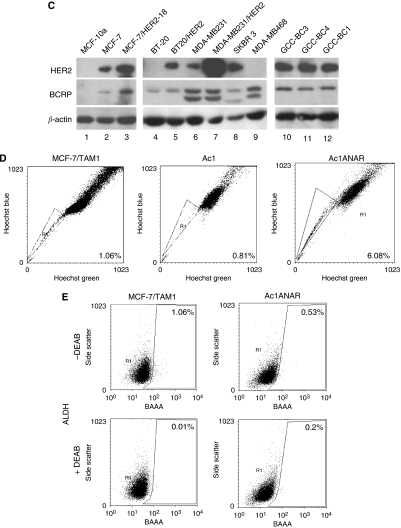
Human epidermal growth factor receptor 2 (HER2) expression and side-population (SP) cells: (**A**) The SP was analysed in various breast cancer (BC) cell lines. After staining with H33342, Hoechst Blue and Hoechst Green were measured using a BD LSR I. The cell population that disappeared in the presence of 1 *μ*M of Ko143 was identified as SP cells. (**B**) A Spearman's rank correlation test was performed for the BC cell lines listed in Table 1. The BC cell lines are ranked #1 for being highest in HER2 expression or SP population and #18 for being the lowest, and then the rank for HER2 expression is plotted to the rank for SP population. Statistical analysis shows a significant correlation between the ranks (*r*^2^=0.75, *P*=0.0003). (**C**) HER2 and breast cancer-resistance protein (BCRP) in BC cell lines were detected by western blot analysis. BCRP and HER2 were detected by immunoblotting with anti-BCRP and anti-HER2/neu antibodies. The blot was then probed with anti-*β* actin as a loading control. BCRP can show two distinct bands (antibody from Sigma #B7185) due to differences in glycosylation. (**D**) SP analysis in drug-resistant cells. SP was increased in hormone therapy-resistant BC cells, MCF-7/TAM and Ac1/ANAR. (**E**) ALDH1 analysis in hormone-resistant BC cells. MCF-7/TAM1 and Ac1ANAR contain a ALDH+ population (top panel). Data depicted in A–E are representative of at least three individual analyses.

Table 1 summarises the percentage of SP cells and surface HER2 expression in a panel of BC cells, including recently established primary BC cell lines freshly derived from patient specimens designated as GCC-BC1, -BC2, -BC3, and -BC4 (Supplementary Table S1). The SP in Lu-type MCF-7/HER2-18 cells increased substantially compared with parental and empty vector-transfected MCF-7 cells (Figure 2A, Table 1). Another clone of MCF-7 cells transfected with *HER2*, designated HC7 (established by Dr Brodie), similarly exhibited a substantial increase in SP compared with parental MCF-7 cells (Table 1). In Ba-subtype BC, the SP in BT-20/HER2 cells also increased compared with that in BT-20/pcDNA3. In Bb-subtype MDA-MB-231/HER2 cells, the SP comprised 0.32% of the total population, whereas no SP cells were detected in MDA-MB-231/pcDNA3 (Table 1). Figure 2B depicts a transformation of data in Table 1, shown as a Spearman's rank correlation of SP and HER2 expression, suggesting a strong correlation (*r*=0.75, *P*=0.0003) of the rank of HER2 expression with the rank in percentage of SP cells.

### HER2 expression and expression of BCRP and Pgp in BC cells

As BCRP is transcriptionally upregulated by HER signalling (Meyer zu Schwabedissen *et al*, 2006), we investigated the effect of HER2 on SP cells in different subtypes of BC cells using reverse transcriptase PCR and western blot analysis. The BCRP mRNA expression was 5.5-fold increased in MCF-7/HER2-18 cells compared with that in parental cells (data not shown; Fisher *et al* (2005); Nakanishi *et al* (2003)) and this translated into a similar increase in BCRP protein expression in these isogenic cell lines (Figure 2C). A small increase in BCRP expression was observed in BT-20 and MDA-MB-231 cells transfected with HER2 when corrected for expression of *β*-actin (Figure 2C). In patient-derived human BC cells, significant expression of BCRP was detected (Figure 2C).

As a relatively large SP was seen in Lu and Lu-like-subtype BCs, we tested different inhibitors of ABC transporters, for example, verapamil (50 *μ*M), FTC (10 *μ*M), and Ko143 (1 *μ*M), to identify specific ABC transporters that function in SP cells (Goodell *et al*, 1996; Allen *et al*, 2002). In most cell lines tested, SP cells were completely and consistently diminished by addition of the specific BCRP inhibitor Ko143 (Figure 2A), but not by the Pgp inhibitor verapamil or FTC (data not shown), suggesting that BCRP has a dominant role in defining SP cells in BC. To assess whether the treatment of Ko143 would also affect CD44^+^/CD24^−^ or ALDH^+^ T-IC fractions, we analysed these markers in MCF-7/HER2-18 cells under the same conditions used for SP experiments. As shown in Supplementary Figure S2A, Ko143 moderately decreased the CD44^+^/CD24^−^ population, but showed an increase in the ALDH^+^ fraction (data not shown), suggesting that the BCRP inhibitor may have no specific effects on these T-IC markers.

### Relationship between hormone resistance and SP cells

**Figure 3:**
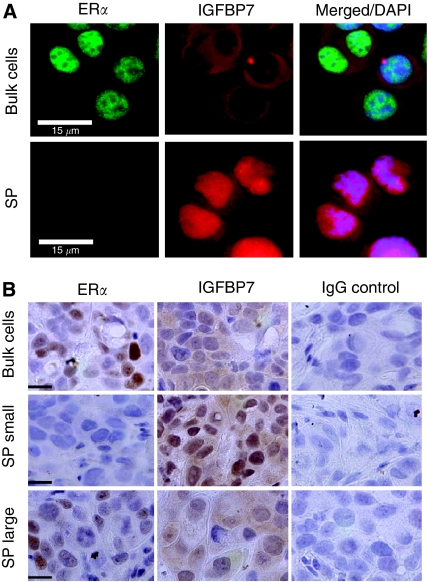
Characteristics of side-population (SP) cells. (**A**) Characteristics of SP in MCF-7/human epidermal growth factor receptor 2 (HER2)-18 breast cancer (BC) cells. The expressions of ER*α* (fluorescein isothiocyanate (FITC) green, left panel) and insulin-like growth factor binding protein 7 (IGFBP7) (TRITC red, middle panel) are shown by immunofluorescence staining; whole cell population cells are compared with SP cells. The right panel shows the merged images using 4′-6-diamidino-2-phenylindole (DAPI) counter staining (blue) to contrast the nuclei. Control cells were probed with mouse and rabbit IgG (Santa Cruz Biotechnolog Inc.). Results represent three independent experiments. White bars represent 15 *μ*m. (**B**) The expressions of ER*α* and IGFBP7 were also compared in tumours established from the whole cell population or from the SP in non-obese diabetic severe combined immunodeficiency (NOD/SCID) mice. Shown are expression levels of the protein in small (100 mm^3^) and large (1500 mm^3^) size grafts arising from 500 SP cells from MCF-7/HER2-18 in NOD/SCID mice. Results represent three independent experiments. Black bars represent 15 *μ*m.

To investigate the impact of hormonal therapy, particularly hormone resistance on BC T-ICs, SP cells were analysed in acquired tamoxifen-resistant MCF-7/TAM1 cells, aromatase-overexpressing Ac1 cells, and the acquired aromatase inhibitor anastrozole-resistant line, Ac1ANAR. It is noteworthy that MCF-7/HER2-18 cells are intrinsically resistant against tamoxifen. As shown in Figure 2D and Table 1, the percentage of SP in MCF7/TAM1 and Ac1ANAR was increased compared with that in MCF-7 and Ac1 parental cell lines. The fraction of ALDH^+^ cells increased to a similar extent as in MCF-7/HER2-18 (Table 2, Figure 3E) compared with parental MCF-7 cells (Figure 1A). CD44^+^/CD24^−^ analysis showed different results; tamoxifen-resistant MCF/TAM1 cells had a four-fold higher CD44^+^/CD24^−^ expression compared with MCF-7, whereas anastrozole-resistant cells had a five-fold lower surface CD44^+^/CD24^−^ expression (Table 2).

### Characterisation of SP in BC

The SP cells isolated from MCF-7/HER2-18 and GCC-BC4 cell lines (Table 1) were subjected to immunofluorescence staining for BC T-IC markers ER*α* and insulin-like growth factor binding protein 7 (IGFBP7) (Shipitsin *et al*, 2007). ER*α* was not detected in the SP of MCF-7/HER2-18 cells, whereas it was detected in the nuclei of cells in the whole cell population (Figure 3A). In contrast, IGFBP7 was localised in the nuclei of SP cells but displayed only weak cytoplasmic staining in the whole cell population (Figure 3A).

The whole cell population was used as control for SP cells instead of isolated non-SP cells because of the cytotoxic effects of H33342 on cells that uptake the dye (Montanaro *et al*, 2004).

However, clonogenic assays using a whole cell population treated with H33342 under conditions of the SP assay (5 *μ*g ml^–1^=8.1 *μ*M) showed that putative T-ICs (colony-forming units) remain viable (Supplementary Figure S2B) to >50%.

To examine the self-renewal properties of SPs, 100 and 500 SP cells were injected into the mammary gland of NOD/SCID mice by limited dilution technique, and tumour growth and histology were compared with injection of 5 × 10^6^ unsorted/bulk cells. Tumours with sizes smaller than 100 mm^3^, formed from SP cells of both MCF-7/HER2-18 (Figure 3B) and GCC-BC4 (Supplementary Figure S2) lines, were negative for ER*α* and positive for nuclear IGFBP7, consistent with the localisation of these markers in SP, as shown in Figure 3A. Tumours of a larger size (≅1500 mm^3^) were well differentiated and developed stroma and blood vessels, similar to tumours established from bulk cells or from the original patient tumour of GCC-BC4. These tumours did express nuclear ER*α* and had lost their nuclear IGFBP7 expression (Figure 3B, Supplementary Figure S3B; Sausville and Burger (2006)). The BCRP membrane positivity was increased two- to three-fold in SP cells, compared with non-SP cells, in both MCF-7/HER2-18 and patient-derived GCC-BC4 cells (Supplementary Figure S1D). In contrast, Pgp expression remained unchanged in both fractions in MCF-7/HER2-18 (data not shown).

### HER2 inhibitors can reduce the SP and prevent the engraftment of breast T-ICs in SP cells

**Figure 4:**
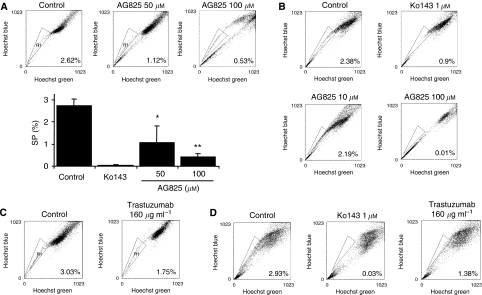

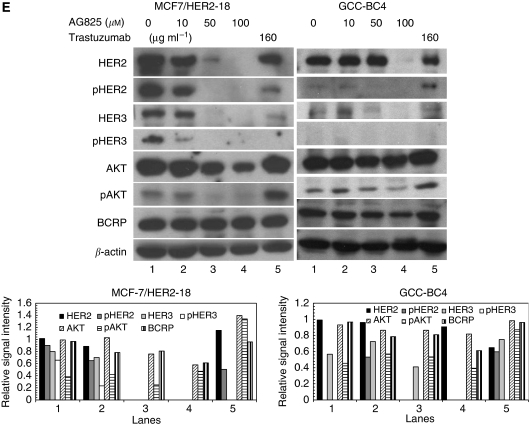
Effect of inhibition of human epidermal growth factor receptor 2 (HER2)/HER3 on side-population (SP) cells. (**A**) Effects of HER2 inhibition by AG825 on SP in MCF7/HER2-18 cells. MCF-7/HER2-18 cells were treated with dimethyl sulphoxide (DMSO) (vehicle), or with 50 or 100 *μ*M AG825 for 72 h. Thereafter, SP cells were analysed as described. Top panels show typical SP cell analysis with % SP in the lower right corner of each panel and the bar graph summarises the effects of HER2 inhibitor on SP in MCF-7/HER2-18 cells. Each bar represents the mean value of at least three independent experiments with s.e.m. ^*^P<0.05, ^**^P<0.01 by Student's *t*-test. (**B**) Effect of AG825 on SP cells in GCC-BC4 cells. Cells were treated with DMSO (vehicle), or with 10 or 100 *μ*M of AG825 for 72 h. The SP cells were analysed by measuring Hoechst Blue and Green fluorescence on a flow cytometer. Experiments were repeated at least three times (% SP is indicated in the lower right corner). (**C**) Effects of HER2 inhibition by trastuzumab on the SP in MCF7/HER2-18 cells. (**D**) Effect of trastuzumab on SP cells in GCC-BC4. K0143 was used as a specific inhibitor of SP cells expressing breast cancer-resistance protein (BCRP) and to set the appropriate gates for the detection of the SPs. (**E**) Comparison of HER2, phosphorylated HER2, HER3, phosphorylated HER3, AKT, phosphorylated AKT, and BCRP expression in MCF-7/HER2-18 and GCC-BC4 cells after treatment with the HER2 inhibitor AG825 at different doses (as indicated) and with trastuzumab (160 *μ*g ml^–1^) for 72 h. Experiments were repeated at least three times. Absolute intensities of each signal were quantified using NIH Image J software, and then a relative intensity value was obtained by dividing the absolute intensity of a given protein by that of *β* actin. Signal quantifications for each lane of the western blots are shown as bar graphs below.

MCF-7/HER2-18 or GCC-BC4 cells were treated with various concentrations of tyrphostin AG825, an experimental inhibitor of HER2. As 50 *μ*M of AG825 abolishes phosphorylation of HER2, but not of HER1, in human lung cancer cells (Fernandes *et al*, 1999), MCF-7/HER2-18 and GCC-BC4 cells were exposed to 50 *μ*M (the estimated IC_50_ value in a 5-day MTT growth assay, Supplementary Figure S2C; Phatak *et al*, (2007)) and other concentrations of AG825 for 72 h. Although 100 *μ*M of AG825, the approximate IC_90_, did not achieve net cell kill in a 5-day growth assay (Supplementary Figure S2C), it drastically reduced SPs in GCC-BC4. AG825 significantly reduced SP cells in a dose-dependent manner in both cell lines that were analysed (Figure 4A and B). Statistical analysis of the SP population in the presence of 50 (*P*<0.05) and 100 *μ*M (*P*<0.01) of AG825 in MCF-7/HER2-18 cells revealed a significant decrease in the percentage of SP cells to 36.9 and 18% of control, respectively (Figure 4A). Statistically significant decreases in SP in response to 3-day treatment with AG825 were also observed in GCC-BC4 cultures (Figure 4B). The average of three independent analyses of the percentage of SP in GCC-BC4 cells treated with 10 *μ*M of AG825 was 67.3% of that in control, whereas 100 *μ*M of AG825 almost completely abolished the SP (*P*<0.01).

Treatment with trastuzumab (160 *μ*g ml^–1^, a therapeutically achievable plasma concentration) for more than 5 days decreased SP to 57% of that in control cells in MCF-7/HER2-18 cells and to 47% in GCC-BC4 cells (Figure 4C and D).

To verify that the decrease in SP seen with AG825 or trastuzumab was mediated by inhibition of HER2 signalling, their effects on phosphorylation status of HER2, HER3, and AKT (a downstream effector of HER signalling) were studied. In MCF-7/HER2-18 cells treated with AG825 for 72 h, phosphorylated HER2 (pHER2) decreased (starting at 10 *μ*M), and was completely abolished at 50–100 *μ*M concentrations (Figure 4E). Concomitantly, pHER3 was reduced in the cells. Expression of pAKT was markedly decreased by AG825 treatment in a concentration-dependent manner. Similar observations were made in GCC-BC4 cells treated with AG825. The effect of AG825 on pHER3 was not clearly detected because its overall expression was very low in these cells. Interestingly, trastuzumab (160 *μ*g ml^–1^) did not reduce pAKT in either cell line, although it did reduce HER2 and HER3 phosphorylation in MCF-7/HER2-18 cells (Figure 4E left). To underline the importance of HER2 over HER1 signalling in relation to a detectable SP in BC cells, we also used the selective HER1 (ErbB1, EGFR) inhibitor AG1478, which has a 100-fold higher specificity for HER1 than HER2 (Egeblad *et al*, 2001). AG825, but not AG1478, was able to downregulate HER2 in MCF-7/HER2-18 cells at their IC_90_ concentrations of 100 *μ*M and 10 *μ*M (Supplementary Figure S2D, S4A). pHER was completely inhibited by AG825, but only to ∼50% by AG1478. As a result, SP was markedly reduced by AG825 only, and to a lesser extent by AG1478 (Supplementary Figure S4C). Moreover, in contrast to AG825, AG1478 did not downregulate BCRP expression (Supplementary Figure S4B *vs* Figure 4E), but inhibited p38 MAPK activation (Supplementary Figure S4B), whereas AG825 did not (data not shown).

Collectively, these observations suggest that the inhibition of HER2 phosphorylation by AG825, resulting in cessation of the *trans* phosphorylation of HER3, and thus complete blockage of HER2 signalling through AKT, might be the predominant factor contributing to the reduction of BC T-ICs seen in SP and tumour repopulation assays. Treatment with up to 100 *μ*M AG825 reduced BCRP expression by about 40% in each cell line, whereas BCRP expression seemed to be unaltered after trastuzumab treatment (Figure 4E).

To assess whether HER2 has a role in the self-renewal of T-ICs in the SP *in vivo*, SP cells from MCF-7/HER2-18 and GCC-BC4 cells were either incubated for 2 h with 100 *μ*M of AG825, or with vehicle control, and then mixed with an equal volume of Matrigel and immediately injected at various dilutions (100,500, or 1000 cells per injection) into the inguinal mammary gland (#4) of NOD/SCID mice. Trastuzumab was used as systemic treatment and injected intraperitoneally at 8 mg kg^–1^ every 4 days until the experiment was terminated. As few as 100 vehicle-treated MCF-7/HER2-18 SP cells formed tumours in NOD/SCID mice with a total tumour incidence of 29 out of 35 injections (Table 3), whereas the engraftment efficiency of the whole cell population at 1000 or 10 000 was 0 takes for every three injections, and at 5 × 10^6^ was three takes for every three injections into the mammary gland. If SP cells were pretreated with AG825, they were unable to repopulate tumours (0 out of 18, *P*<0.001). When the animals received intraperitoneally trastuzumab, tumours repopulated from SP cells in only 2 out of 16 cases (*P*=0.02) (Table 3). When untreated GCC-BC4 SP cells were injected, all control SP cells (8 out of 8) grew tumours. When these SP cells were pretreated with AG825, only one out of eight injections maintained repopulating capacity (1 out of 8, *P*<0.001) (Table 3). Impairment of the engrafting capability of BC T-ICs by blocking HER2 signalling may in part account for the remarkable efficacy of trastuzumab in preventing recurrence in the adjuvant treatment setting in BC.

## Discussion

Although a regulatory role for HER2 was suggested in ALDH1^+^ T-ICs, to date, no information is available regarding HER2 expression in SP cells, a T-IC-enriched cell fraction key to therapy resistance. Wicha and co-workers have reported that ALDH1^+^ T-ICs are more frequently detected in receptor-negative basal subtypes of BC cell lines (16 out of 16 cells were positive for ALDH1) than in luminal subtypes (5 out of 12 cells were positive) (Charafe-Jauffret *et al*, 2009). The latter study did focus on ALDH1^+^ BC cell types only and did not include a comparison of HER2 and ALDH1 expression with other known T-IC markers, CD44^+^/CD24^−^ or SP fraction. Thus, there is a paucity of data describing T-IC markers in different subtypes of BC.

We have examined the occurrence of SP and the effects of HER2 on SP among receptor-negative BC cell lines (Ba and Bb subtypes) and luminal cell lines (Lu subtype) that were also characterised for ALDH^+^ and CD44^+^/CD24^−^ expression. We observed that breast T-IC markers differ by transcriptional gene expression profiles (Neve *et al*, 2006), as shown in Figure 1 and Table 2; SP, CD44^+^/CD24^−^, and ALDH1^+^ cell fractions vary among Lu, Ba, and Bb BC cells. Whereas SP is most frequent in Lu subtypes, Bb cells are characterised by CD44^+^/CD24^−^T-ICs, although they lack SPs. Interestingly, the association of SP with the Lu-subtype gene cluster was confirmed by our primary patient cell cultures, which were all ER+ and expressed measurable amounts of HER2 (see Supplementary Table S1), but further studies with primary tissues must confirm the association of CD44 positivity and high activity of ALDH1 expression with basal-subtype cancers. If confirmed in a larger cohort of primary cancers, the existence of distinct types of T-ICs in BC will have consequences for targeting such cells with drugs. For example, the Lu-subtype BC T-ICs should be more susceptible to clinically available anti-HER2 therapies such as trastuzumab or lapatinib.

We report here for the first time that HER2 expression increases breast T-ICs in SP in a variety of human BC subtypes (Figure 2C, Table 1). HER2/HER3 signalling may also have a role in modulating the SP mainly through AKT (Figure 4E). Although previous studies have reported an increase in SP cells by activation of HER1/EGFR signalling (Chen *et al*, 2006), and a stimulatory effect of EGF on BCRP gene transcription (Meyer zu Schwabedissen *et al*, 2006), experiments conducted by us comparing the effects of the HER2 inhibitor AG825 with that of the HER1 inhibitor AG1478 (Figure 4 and Supplementary Figure S4) demonstrate a predominant role of HER2/HER3 signalling over HER1 signalling in regulating the SP. HER1 inhibition and, thus, cessation of MAPK signalling could not reduce BCRP expression in GCC-BC4 cells despite inhibition of phospho p38 MAPK (Supplementary Figure S4B). In addition, AG1478 did not inhibit HER2 expression and only moderately affected pHER2 expression (Supplementary Figure S4A). This resulted in only limited effects of AG1478 on the SP (Supplementary Figure S4C).

The importance of HER2 expression in the occurrence of a large SP is further supported by our data resulting from the analyses of hormone therapy-resistant BC cell lines and tumours. It has been shown that HER2 overexpression may have a role in developing resistance to the aromatoase inhibitor letrozole and that tumours relapsing early while on adjuvant tamoxifen therapy exhibit high levels of HER2 protein and/or gene amplification (Shin *et al*, 2006). This may be reflected in the increased SP and HER2 expression data for tamoxifen-resistant cell lines MCF-7/HER2-18 and MCF-7/TAM1, and the aromatase inhibitor anastrozole-resistant cell line Ac1ANAR (Figure 2D–E, Table 1). In fact, Brodie and colleagues have shown that trastuzumab can reverse letrozole resistance by restoring oestrogen receptor function and sensitivity to oestrogen (Sabnis *et al*, 2009). However, in the letrozole-resistant LTLT-Ca cell line, HER2 inhibition by trastuzumab affected pMAPK rather than pHER2. This could explain the discrepancy seen in our experiments with trastuzumab. The latter was unable to inhibit pAKT, and pHER2 was affected only to a limited extent (Figure 4E); thus, the inhibition of SP was moderate, as seen for the MAPK inhibitor AG1478 signalling through pMAPK (Supplementary Figure S4).

Emerging evidence suggests that the HER2/AKT pathway regulates T-ICs in BC by activating the Wnt pathway (Korkaya *et al*, 2009); however, a precise mechanism of the manner in which Wnt signalling contributes to the BC T-IC phenotype requires further investigation.

Analysis of the hormone-resistant cell lines for MCF-7/HER2-18, MCF-7/TAM1, and Ac1ANAR for CD44/CD24 and ALDH expression indicated that CD44^+^/CD24^−^ is doubled to quadrupled in tamoxifen-resistant lines, but not in aromatase inhibitor-resistant lines (Figure 2D–E, Table 2). A similar trend was seen for ALDH1; however, the levels remained generally lower than in basal-type BCs.

A genomic profiling analysis of isolated CD44^+^ (stem/progenitor cells) and CD24^+^ (mature cells) populations from normal and invasive primary breast tissues (Shipitsin *et al*, 2007) found a panel of four genes that were critical differentiation markers, including HER2 and ER, as well as seven genes that were associated with stem cell characteristics such as IGFBP7. The loss of ER*α* expression in SP cells or in small transplanted tumours (Figure 3, Supplementary Figure S3B) is consistent with this report by Polyak and co-workers who found ER*α* downregulated in CD44^+^ compared with CD24^+^ cells (Shipitsin *et al*, 2007). However, in our studies, ER*α* was re-expressed, as the T-IC-derived SP tumours underwent differentiation and growth (Figure 3B, Supplementary Figure S3B). Moreover, IGFBP7, initially identified by us as a protein that is downregulated with disease progression in BC (Burger *et al*, 1998; Landberg *et al*, 2001), was described as a stem cell marker expressed in CD44^+^, but not in CD24^+^ cell fractions of normal and BC tissues (Shipitsin *et al*, 2007). When we stained SP cells, SP-derived small tumours, large differentiated tumours, and tumours established from bulk cells, we found that IGFBP7 was very weakly expressed and mainly present in the cytoplasm of differentiated/bulk tumour cells, but was very strongly expressed in the nucleus of SP cells (Figure 3B, Supplementary Figure S3). This is also consistent with other stem cell literature reporting IGFBP7 as a gene that is downregulated in differentiation pathways (Bruno *et al*, 2004) and being one of the most highly expressed genes in embryonic stem cells (Liu *et al*, 2006). The redistribution of IGFBP7 from the cytoplasm into the nucleus and its upregulation indicate a possible transcriptional function in T-ICs. Together, the IGFBP7 and ER data and the tumour repopulation assays confirm the stem cell-like properties of cells in the SP.

In this study, BCRP was detected by western blot in three of the patient-derived cell lines (GCC-BC1, -BC3, and -BC4, Figure 2C) and BCRP expression was higher in SP than in non-SP cells in GCC-BC4 cells (Supplementary Figure S1D). As expression of BCRP is transcriptionally regulated by HER1/HER2-mediated signalling, the detailed mechanism of transcriptional regulation of BCRP in T-ICs has to be clarified. Alternative promoter usage was reported in BCRP transcription (Nakanishi *et al*, 2006a). Therefore, increased expression of BCRP could be subjected to alternative promoter usage in response to transcriptional regulators specific to T-ICs in the SP. Thus, the expression of BCRP might be an important property of SP in human BC and may contribute to drug resistance of T-ICs in the SP. Moreover, if drug-resistant T-ICs lead to treatment failure, their simple persistence in a protected stem cell ‘niche’ environment throughout the course of chemotherapy could lead to repopulation of the tumour after its initial successful debulking, owing to chemoresponsive, mature tumour cells. This may explain why in studies examining BCRP expression, increased ABC transporter levels were not seen in BC patients who received only three cycles of preoperative anthracycline-based therapy compared with chemo-naïve patients (Faneyte *et al*, 2002). Hence, to determine a role of BCRP and the SP in the resistance of BC to chemotherapy, BCRP expression must be determined in relapsed tumours.

In summary, our data provide the first evidence that SP is enriched in T-ICs in the luminal subtype of BC, and that HER2 expression has a pivotal role in expansion of the luminal-type BC T-IC population. As the SP in BC is identified by functional expression of multidrug-resistant proteins, in particular BCRP, our findings may present a new rationale for the poor response of HER2-positive and hormone-resistant BC to conventional chemotherapeutics. Therefore, HER2 alone or the HER2/HER3 signalling pathway could be effective targets to modulate or reverse drug resistance of T-ICs in luminal subtypes of BC, resulting in suppression of reoccurrence or metastasis of BC.

## Figures and Tables

**Table 1:** SP cells and HER2 expression in human breast cancer cells

			**SP**		**HER2**	
**Cell line isogenic subclone**	**ER*α***	**Gene cluster**	**Rank**	**(%)**	**Rank**	**MFI**
MCF10A	−	Bb	1	0.00 (0.00, 0.00)	2	0.70±0.13
MCF-7	+	Lu	7	0.39±0.15	9	40.2±11.8
MCF-7/vector	+	Lu	—	0.29±0.21	—	ND
MCF-7/TAM1	+	Lu	10	0.87±0.27	10	40.9±6.30
MCF-7/HER2-18	+	Lu	16	3.38±0.27	17	161.4±37.4
HC7	+	Lu	18	7.31±0.55	18	170.3 (97.48, 247.16)
T47D	+	Lu	4	0.15±0.08	5	19.2±6.19
Ac1	+	Lu	11	1.05±0.32	15	125.2±40.5
Ac1ANAR	+	Lu	17	6.00±0.39	14	84.9±25.8
SKBR3	−	Lu	12	1.41±0.27	16	156.1±83.88
BT-20	−	Ba	−	0.10±0.04	−	ND
BT-20/pcDNA3	−	Ba	5	0.17±0.08	4	7.30±1.70
BT-20/HER2	−	Ba	8	0.76±0.33	12	48.5±8.27
MDA-MB-468	−	Ba	1	0.00±0.00	1	0.00±0.00
MDA-MB-231/pcDNA3	−	Bb	1	0.00±0.00	3	1.50±0.84
MDA-MB231/HER2	−	Bb	6	0.32±0.12	13	82.6±14.5
GCC-BC1	+	Lu-like	14	2.92±1.26	7	31.6±9.09
GCC-BC2	+	Lu-like	15	3.15±1.97	11	45.0±8.32
GCC-BC3	+	Lu-like	9	0.85±0.33	6	25.4±8.39
GCC-BC4	+	Lu-like	13	2.80±0.25	8	37.6±10.8

Abbreviations: ANAR=anastrozole resistant; TAM=tamoxifen resistant; ND=not done; MFI=mean fluorescence intensity; SP=side population; HER2=human epidermal growth factor receptor 2 ER*α*=estrogen receptor-*α*.

Each value represents the mean value of three independent experiments with±s.e. When an experiment was performed only twice, each value is shown in parentheses. A rank of 1 represents the lowest value for SP and HER2 expression.

**Table 2:** Stem cell populations in breast cancer cells

		**SP**	**CD44^+^/CD24^−^**	**ALDH^+^**
**Cell line**	**Gene cluster**	**(%)**	**(%)**	**(%)**
MCF-7	Lu	0.39±0.15	4.80±0.49	0.33±0.19
MCF-7/HER2-18	Lu	3.38±0.27^**^	7.36±3.03^*^	1.17±0.27^*^
MCF-7/TAM1	Lu	0.87±0.27	20.25±0.23^**^	0.82±0.26^*^
Ac1/ANAR	Lu	6.00±0.39^**^	0.875±0.8	0.45±0.07
MDA-MBA-468	Ba	0.00±0.00	0.29±0.17	7.56±1.26
MDA-MB-231	Bb	0.00±0.00	80.3±3.86	4.42±0.34
GCC-BC4	Lu-like	2.02±0.95	1.68±0.94	1.02±0.36

Abbreviations: SP=side population; ALDH=aldehyde dehydrogenase; ANAR=anastrozole resistant.

^*^P<0.01 and ^**^P<0.05 *vs* MCF-7 by Student's *t*-test.

Tumour-initiating cell markers are shown as mean values±s.e. Experiments were independently repeated for three times.

**Table 3:** NOD/SCID mouse repopulation assay

		**Control**	**AG825**	**Trastuzumab**
**SP cell type**	**SP cells injected**	**Tumours/injections**		
MCF-7/HER2-18	100	11/14	0/7	0/5
	500	10/13	0/9	0/8
	1000	8/8	0/2	2/3
GCC-SC-4/Patient	100	4/4	1/4	—
	500	4/4	0/4	—
Total		37/43a,^b^	1/26a,^b^	2/16a,^b^

Abbreviations: NOD/SCID=non-obese diabetic severe combined immunodeficiency; SP=side population.

a Comparison within groups: control, *P*>0.7; AG825, *P*<0.001; trastuzumab, *P*=0.02.

b Comparison between groups: control *vs* AG825, *P*<0.001; Control *vs* trastuzumab, *P*=0.002. The analysis of variance *F*-test was used.

## Supplementary Material

Supplementary Informations
