# Supplementary material for: Side-population cells in luminal-type breast cancer have tumour-initiating cell properties, and are regulated by HER2 expression and signalling
Source: Br J Cancer. 2010 Feb 9;102(5):815–26. doi: 10.1038/sj.bjc.6605553 (PMC2833247; doi:10.1038/sj.bjc.6605553)
Supplement: Supplementary Informations [file 6605553x1.pdf]

SUPPLEMENTAL DATA

Supplemental Methods

Establishment of Primary Cultures from Patient Samples. Fresh breast tumors were received within one hour after surgery from patients who gave informed consent according to procedures approved by the University of Maryland Institutional Review Board. The tumors were sterilely minced in Iscove's Modified Dulbecco's Medium (IMDM, Sigma-Aldrich, St. Louis, MO) medium to yield less than 2x2-mm pieces and cultured in IMDM containing 20% FBS and 1% antibiotic-antimycotic (Invitrogen, Carlsbad, CA) at $37^oC/5\% CO_2$. Attached cells were propagated after 2-3 weeks. Cells were used and analyzed within the first 8 passages and sporadically at higher passages (upto P16). HER2 and estrogen receptor (ER) status were analyzed by immunocytology and real-time PCR (polymerase chain reaction). A list and characteristics of the established patient lines are provided in supplemental table S1.

Quantitative RT-PCR. RT-PCR was performed for BCRP and HER2 as previously described (Nakanishi et al., 2003). For HER2, the primer sequences were as follows: 5'-GACCCGCTGAACAATACC-3' (sense) and 5'-CGGGAGCCCTTACACAT-3' (antisense). The expression was normalized by hypoxanthine phosphoribosyl transferase mRNA expression as previously described (Fischer *et al*, 2005).

MTT Assay. The MTT proliferation assay was performed as described by us in detail before (Phatak *et al*, 2007). Inhibitory concentrations 50%, 90% and 100% were determined based on actual growth inhibition compared to cell growth at the time of drug addition, following the criteria for the interpretation of *in vitro* cyotοtxicity data as established by the US National Cancer Institute (http://dtp.nci.nih.gov/branches/btb/ivclsp.html).

Supplemental Results

SP Analysis by BD LSR I and FACSVantage SE. According to Goodell *et al.* (Goodell *et al*, 1996), the SP is defined as a cell fraction with lower intracellular accumulation of H33342 when it is excited by UV laser at 350 nm and its fluorescence is measured with a 450/20 filter (Hoechst Blue, on the vertical axis) and a 675 EFLP optical filter (Hoechst Red, on the horizontal axis). The low dye accumulation is due to the expression of the ABC transporters including BCRP (ABCG2) and Pgp (ABCB1). The emission peak of H33342 is at 450 nm, but it does not emit fluorescence at > 670 nm. We have analyzed the SP in 4 primary (GCC-BC1-4) and 21 permanent BC cell cultures (**Figure 2A-B and Table I**). We employed two flow cytometers to measure SP cells: first we detected the SP by displaying Hoechst blue and red fluorescence measured by a FACSVantage SE (BD Biosciences); second we employed an LSR I FACS instrument (BD Biosciences) which uses a Helium-Cadmium laser (325 nm) for excitation and detects fluorescence emission using a 424/44 filter (for Hoechst Blue) and a 510/20 filter (for Hoechst green fluorescence – the LSR I cannot detect the red fluorescence). In comparing the two instruments, SP measured by the LSR shown as Hoechst blue vs. green fluorescence profiles was essentially identical to the SP measured as Hoechst blue vs. red fluorescence by the FACSVantage SE (**Figure S1A**). Furthermore, the frequencies of SP cells measured by LSR I and FACSVantage SE were highly correlated in ten different BC cell lines ($R=0.95$, $p=2.6 \times 10^{-5}$). When the SP was further analyzed in MCF-7/HER2-18 and GCC-BC4 (Greenebaum Cancer Center-BC 4) cells with both instruments, the frequency of SP measured by the BD LSR I was approximately equal to that obtained by the FACSVantage SE; 3.34% vs 3.23 % in MCF-7/HER2-18 cells and 2.81% *vs*. 2.91% in GCC-BC4, respectively (Figure S1C). Therefore, we used the BD LSR I for most of the experiments presented here instead of FACSVantage SE, because of easy access and use of the LSR I.

Establishment of Primary Cultures from Patient Samples. A total of 21 breast cancer specimens from mastectomies were received and the establishment of primary cultures attempted. Four specimens were ductal carcinoma *in situ*, one of which grew in culture, but could not be passaged. One specimen was a mucinous adenocarcinoma, 16 were invasive ductal carcinomas. Six invasive ductal carcinomas grew in culture and 4 could be propagated beyond 8 passages. All of those were strongly HER2 positive by flow cytometry and immunocytology (see **Figures S1D, S3A, Table I**). All were also ER+ per pathology report, immunocytology (**Figure S3A, Table S1**) and PCR (data not shown). Their histological grade was 2 or 3 (**Table S1**).
The cell lines grow only in IMDM containing 20% FBS, but not for example in DMEM or lower serum content, suggesting that the medium used for primary culture establishment might have selected for luminal type breast cancers. To assure the genomic stability and identity with the patient tumor from which they were derived for the four cell lines, GCC-BC1, -BC2, -BC3, and -BC4 used in our SP studies, we frequently analyzed ER, HER2 and CK18 expression by immunoflurescence (**Figure S3A**), FACS analysis or PCR. We also compared the histological features of tumors arising from the SP cells or the whole cell populations (injected at $5x10^6$ cells s.c.) to those of the original patient tumor (see e.g. GCC-SC4, **Figure S3B**). This is an accepted method of confirmation that primary cell lines are comparable to the patient tumor from which they originated (Sausville and Burger, 2006).

Figure Legend for Supplemental Figures

Figure S1. Comparison of SP Cells Detected by LSR I and FACSVantage FACS Analyzers. **A.** MCF-7/HER2-18 cells were analyzed for the presence of a SP by using a BD LSR I four-laser flow cytometer, where by H33342 dye is excited by a 325 nm Helium-Cadmium laser and the fluorescence profiles (Hoechst Blue vs. Green) measured at 424/44 nm and 510/20 nm. For comparison the FACSVantage SE machine was used, where Hoechst dye is excited at 350 nm with an Argon laser and fluorescence emission determined for Hoechst Blue at 405/30 and Hoechst Red at 640/30. **B.** Comparison of the % SP cells in MCF-7/HER2-18 and GCC-BC4 cells for LSR I and FACSVantage SE analyzers. No difference was seen. **C.** The correlation coefficient of results for SPs from either FACS analyzer is very high (r=0.95) and thus the results are virtually identical showing that both machines can be used. **D.** BCRP in the SP of MCF-7/HER2-18 and GCC-BC4 cells. The expression of BCRP was analyzed in SP and non-SP cells. SP and non-SP fractions were gated as shown in the left panel. In the right panel, the fluorescence corresponding to BCRP expression was determined in the SP (solid line) and non-SP (dotted line) cells relative to isotype controls (grey line). The histograms are representative of three individual experiments.

Figure S2. Cytotoxicity Assays. A. Effects of Ko143 on the $CD44^{+}/CD24^{-}$ fraction in MCF-7/HER2-18 cells. Cells were treated for 90 min. with vehicle control or 1μm Ko143 prior to staining them with anti-CD44 and CD24 antibodies for FACS analysis. **B.** Clonogenic assay for whole cell population MCF-7/HER2-18 cells treated with vehicle control or 8.1μM H33342 for 90 minutes. Cells were washed twice with PBS before seeding them into soft agar. The number of colonies forming per 10,000 seeded cells is shown. **C.** Five day methyltetrazolium (MTT) proliferation assay results for the treatment of MCF-7/HER2-18 and GCC-BC4 cells with AG825. **D.** Five day MTT assay for the treatment of MCF-7/HER2-18 and GCC-BC4 cells with AG1478. Cells in C and

D were continuously exposed to drug concentrations ranging from 0.1 to 250 μM. The growth inhibitory 50% values forAG825 in both cell lines are approximately 50 μM, the IC90 values are 100 μM, indicating that SP inhibition studies and the NOD/SCID tumor repopulation assays were performed at pharmacological drug concentrations. All data are representative of three independent experiments.

Figure S3. Characterization GCC-BC4 Cells. **A a.** Expression of the epithelial cell marker cytokeratin 18 (CK18) in GCC-BC4 cells in passage 6 from patient tissue. **A b**. Expression of ERα in GCC-BC4 cells as in A.a. **A.c.** Expression of HER2 in GCC-BC4 cells as in A.a. Size bar, 15μm.

B a. Hematoxylin and eosin stained tissue section from the original patient tumor of which the primary cell line GCC-BC4 was derived. **B b.** Hematoxylin and eosin stain section from a tumor that was repopulated by 500 SP cells isolated from a GCC-BC4 primary culture in a NOD/SCID mouse and has high similarity to B a. **B c.** Staining for ERα in GCC-BC4 SP-derived tumors arising in NOD/SCID mice is negative (small size 0.1 cm). **B d.** Staining for IGFBP7 in GCC-BC4 SP-derived tumors arising in NOD/SCID mice is strongly nuclear. Size bar, 20 μm.

Figure S4. Effects of HER2 and HER1 signaling inhibition on the SP. **A.** Western blot showing MCF-7/HER2-18 cells treated with the HER2 inhibitor AG825 and the HER1 inhibitor AG1478 and their effects on HER2 or pHER2 levels. B. Western blot of GCC-BC4 cells treated with the HER1 inhibitor AG1478 and its effects on BCRP or phospho p38 MAP kinase. Beta actin was used as equal loading control. **C.** Comparison of effects of AG825 and AG1478 at their IC_{50}s and IC_{90}s on the SP in MCF-7/HER2-18 cells respectively.

Supplemental Table S1: Characteristics of Patient-Derived Breast Cancer Cell Lines

Cell Line	A	R	S	HP	T	M	N	GR	ER (%)/status	PR (%)/status	HER2 IHC/FISH	Ki67 (%)
**GCC-BC1**	59	H	F	IDC	2	0	1	2	89/+	0/-	2+/no	29
**GCC-BC2**	68	C	F	IDC	2	0	2a	3	NA/+	NA	2+/no	NA
**GCC-BC3**	51	C	F	IDC	2	0	0	2	49/+	0/-	3+/amp	41
**GCC-BC4**	62	AA	F	IDC	1a	0	0	1	67/+	0/-	2+/no	15

A, age; R, ethnicity: H, Hispanic, C, Caucasian, AA, African American; S, sex, F, female; HP, histopathology, IDC, invasive ductal carcinoma; T, tumor size according to the American Joint Committee on Cancer (AJCC) staging system; N, lymph node involvement; M, metastasis; GR, Nottingham grade; ER, estrogen receptor (>10% = positive (+)); PR, progesterone receptor; IHC, HER2 overexpression by immunohistochemistry (IHC) CB11 antibody, FISH, HER amplified (amp) and not amplified (no); Ki67, proliferation index as % positive nuclei; NA, not available.

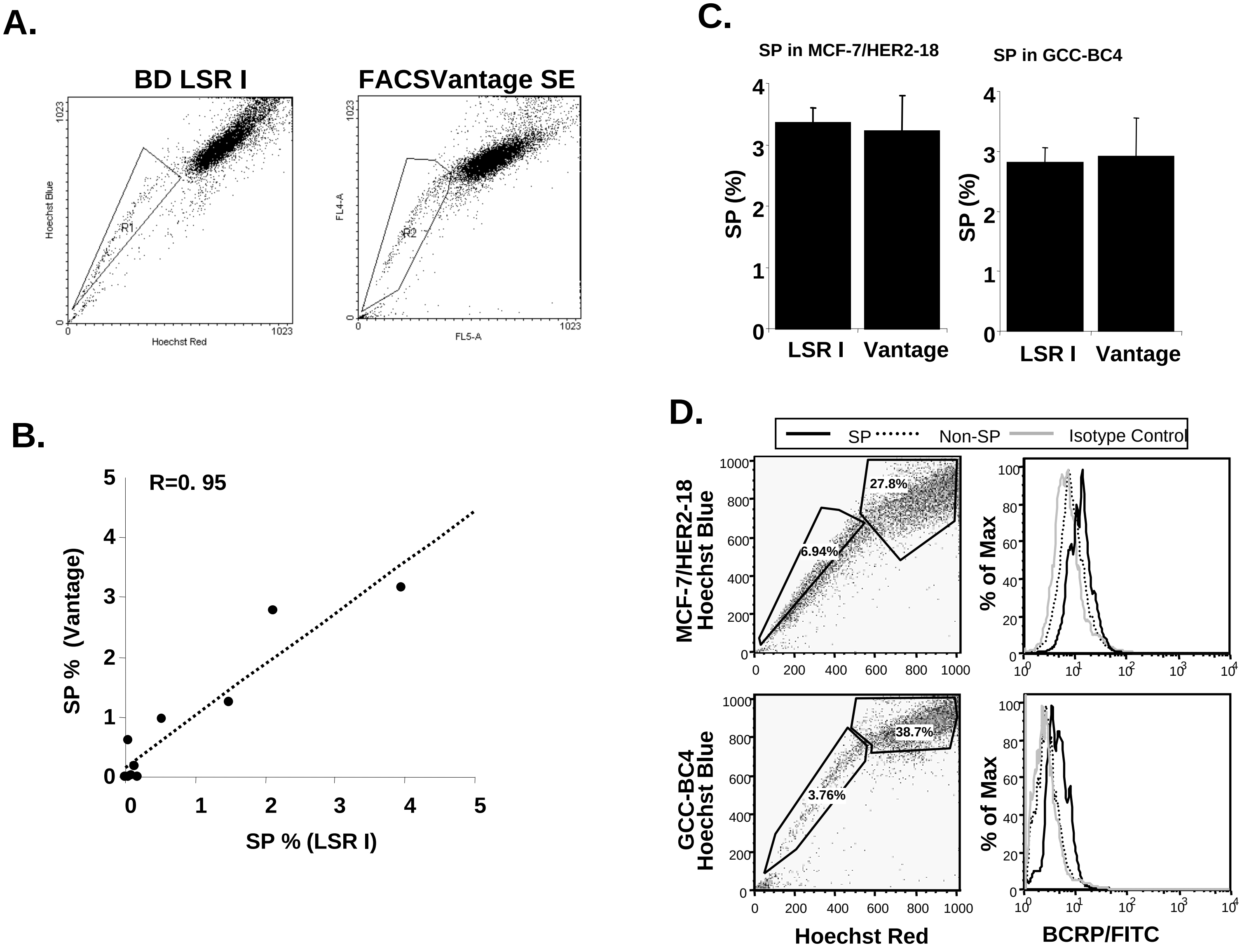

A.
BD LSR I
FACSVantage SE
Hoechst Blue
Hoechst Red
R1
R2
FL4-A
FL5-A
1023
0
B.
R=0. 95
SP % (Vantage)
SP % (LSR I)
0
1
2
3
4
5
C.
SP in MCF-7/HER2-18
SP in GCC-BC4
SP (%)
LSR I
Vantage
D.
SP
Non-SP
Isotype Control
MCF-7/HER2-18 Hoechst Blue
GCC-BC4 Hoechst Blue
27.8%
6.94%
38.7%
3.76%
% of Max
Hoechst Red
BCRP/FITC

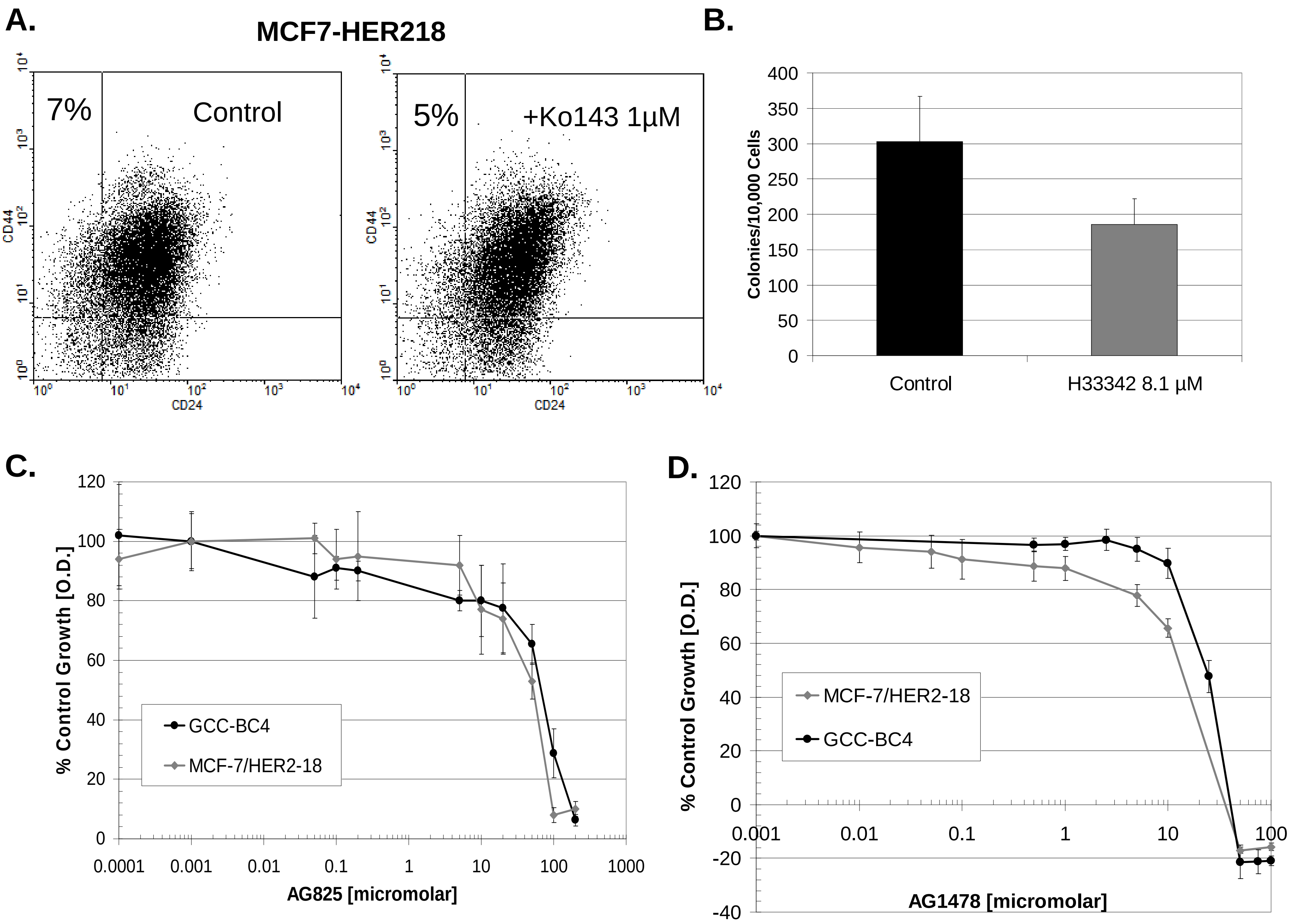
A.
MCF7-HER218
7%
Control
5%
+Ko143 1µM
CD44
CD24
B.
Colonies/10,000 Cells
Control
H33342 8.1 µM
C.
% Control Growth [O.D.]
GCC-BC4
MCF-7/HER2-18
AG825 [micromolar]
D.
% Control Growth [O.D.]
MCF-7/HER2-18
GCC-BC4
AG1478 [micromolar]

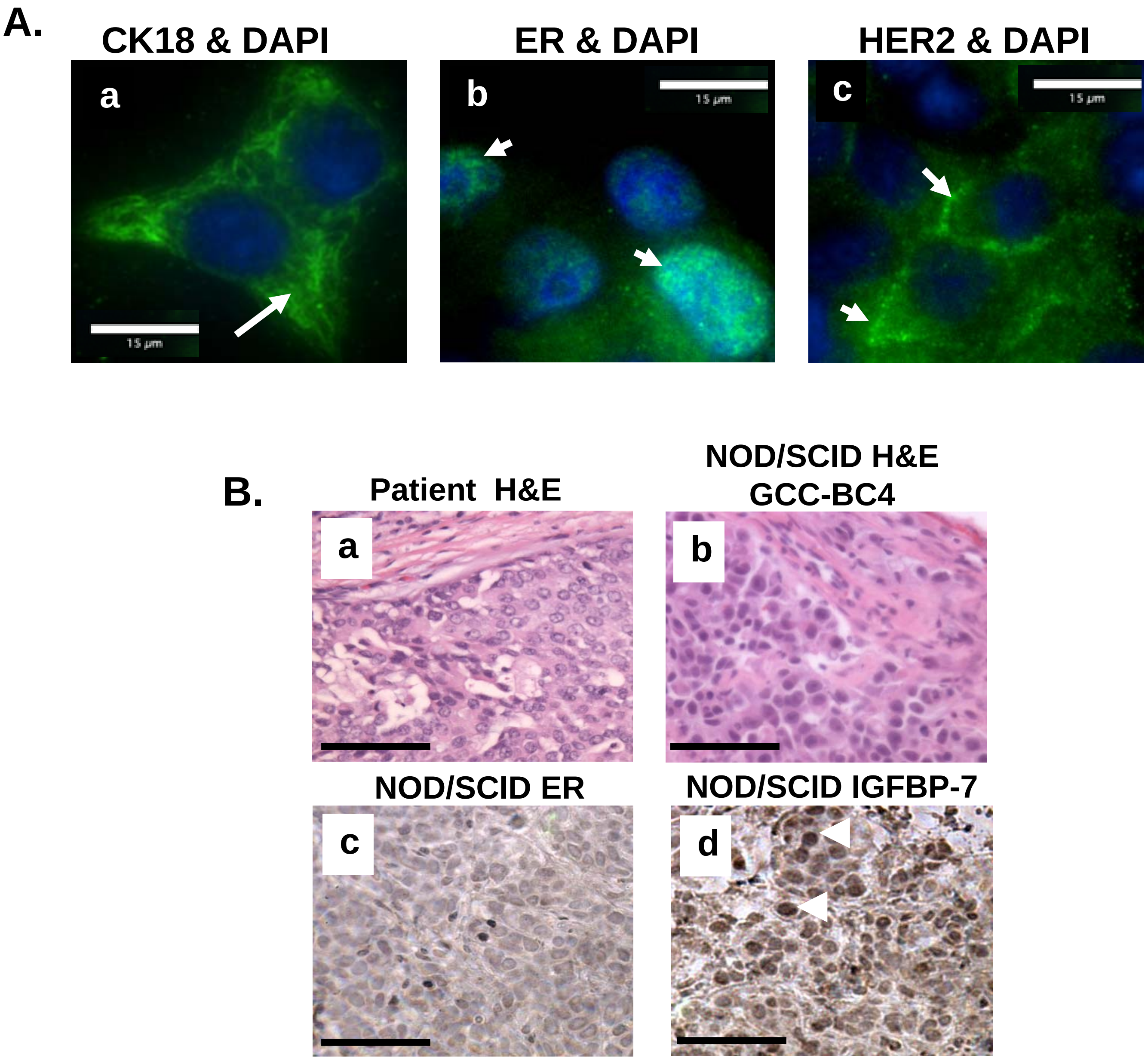
A.
CK18 & DAPI
ER & DAPI
HER2 & DAPI
a
b
c
15 µm
15 µm
15 µm
B.
Patient H&E
NOD/SCID H&E
GCC-BC4
a
b
NOD/SCID ER
NOD/SCID IGFBP-7
c
d

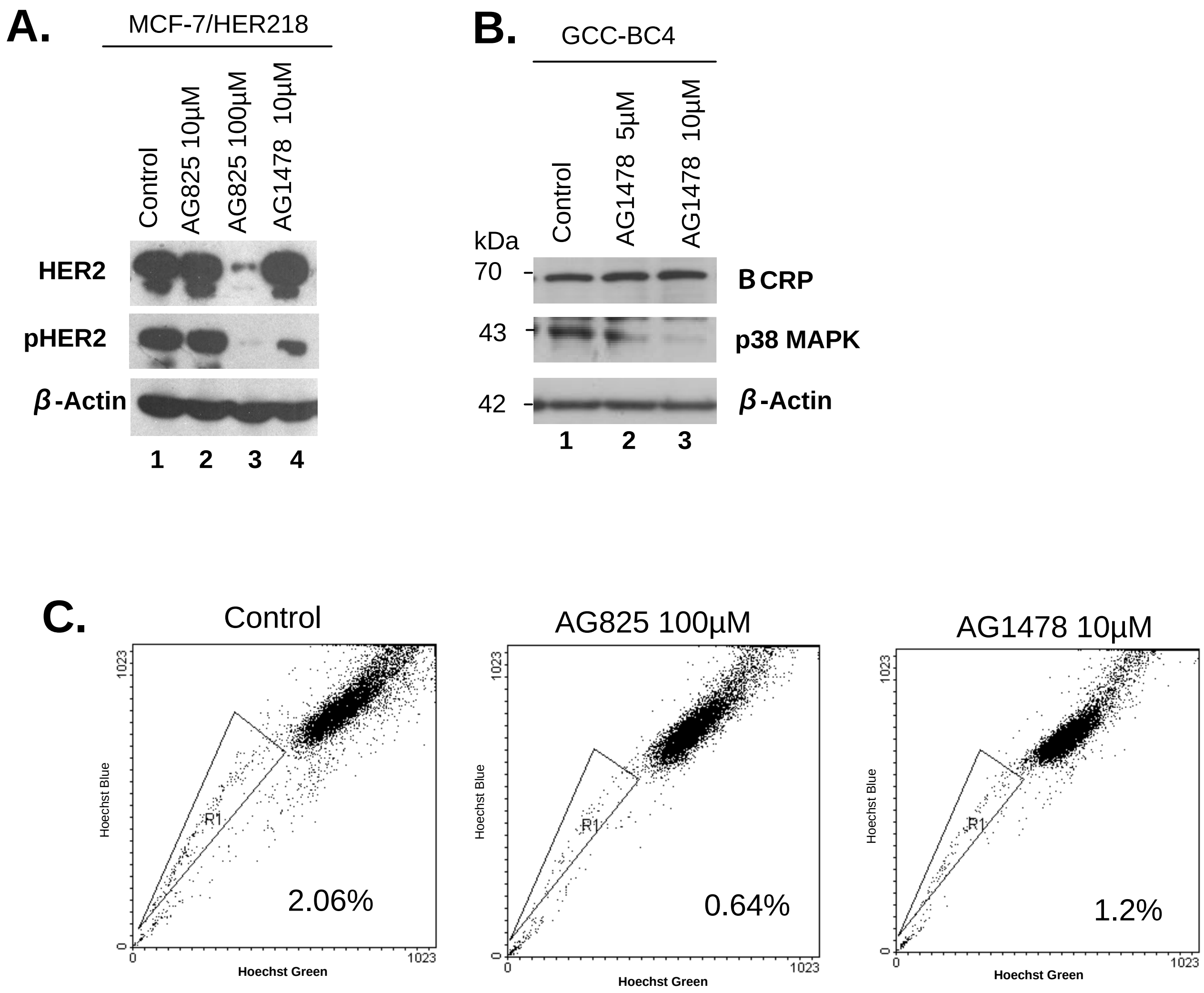

A.
MCF-7/HER218
Control
AG825 10µM
AG825 100µM
AG1478 10µM
HER2
pHER2
β-Actin
1 2 3 4
B.
GCC-BC4
kDa
Control
AG1478 5µM
AG1478 10µM
70
BCRP
43
p38 MAPK
42
β-Actin
1 2 3
C.
Control
AG825 100µM
AG1478 10µM
Hoechst Blue
Hoechst Green
R1
2.06%
0.64%
1.2%
0
1023
